# Black in Marine Science: A new wave is here

**DOI:** 10.1371/journal.pbio.3001833

**Published:** 2022-10-17

**Authors:** Tiara Moore

**Affiliations:** 1 Black in Marine Science, Spokane, Washington, United States of America; 2 The Nature Conservancy, Seattle, Washington, United States of America; Smithsonian Institution, UNITED STATES

## Abstract

After decades of feeling isolated in the field, a group of Black marine scientists and allies came together to create the first ever Black In Marine Science Week. This Perspective discusses how the Black In Marine Science organizing team realized that after the major success of the week and the uncovering of shared experiences of racism, there was much more work to be done.

## Teaser

Black in Marine Science was founded in 2020 to celebrate black marine scientists, spread environmental awareness, and inspire the next generation of scientific thought leaders. Black in Marine Science began as a week but has become a movement.

“Hol up, where the Black in Marine Science Week at…” I tweeted one August afternoon. There had been an increase in Black in STEM weeks [1] due to the lack of diversity and racial injustices in STEM fields, and I had not seen one focused on marine science yet. I never thought that tweet would start a movement that honestly saved my life. At the time, I was a full-time marine scientist with all the credentials, but unfortunately, I still had not “earned my seat at the table.” Marine science is a predominantly white field; >85% of PhDs are awarded to white people in geoscience-related fields annually and <2% awarded to people who identify as black [2]. As such, I never expected the field or spaces I worked in to be filled with black people, but I also did not expect them to be so toxic and unwelcoming [3]. After spending years in school and earning postdocs at prestigious research institutions, I knew I would be respected and welcomed in the field, but sadly found that people did not think I belonged in marine science simply because I was black. I did not believe it at first. I am the baby of almost every diversity initiative that has been launched. Millions of dollars had been spent over the past 40 years with the goal of getting more people from minorities, specifically black people, into marine science, but little to no progress had been made [2], ultimately leading to my tweet.

Within days of the tweet, we created an organizing committee of black marine scientists and allies with the goal of planning the first week dedicated to celebrating black people in marine science. We hosted panels about our research but also racial experiences we had while in the field. We had workshops on coding, networking, and SCUBA diving while taking care of black hair. It was amazing! I was no longer the only black person in the room [3] and I finally had a community of colleagues around the globe, all with similar stories of racism, but still excited about marine science.

After the success of Black in Marine Science (BIMS) Week and raising over $25,000, we decided to form a nonprofit and became incorporated in December 2020. In 2021, we created BIMS TV (Table 1) to not only amplify the voices/research of black marine scientists, but also increase ocean literacy in frontline communities worldwide. To date, we have over 150 episodes, more than 1,600 subscribers and have received 22,000+ views [4]. The goal of BIMS TV is much larger than showing off beautiful black faces who do science; we are providing educational tools that can be used by anyone. BIMS TV is the first step towards educating people about ocean sustainability; we can help the communities that are the most impacted by climate change learn how to mitigate and understand what is happening in their own backyards. Outside of our dedicated online viewing, we have also developed relationships with various schoolteachers who use our videos weekly in their classrooms. With an increase in funding, largely from a grant by The Packard Foundation, we have also been able to pay all our BIMS TV content creators. Not only are we educating people about the ocean, we are increasing the blue economy in my eyes. This helps to dismantle decades of racial wage inequities and the problem of science communication activities going largely uncompensated. We are changing who people see as scientists and how people fundamentally do science in general.

**Table 1:** Black in Marine Science programs.

Program	Description	Schedule
BIMS Bites	The first in our BIMS TV series, these are 5-minute episodes of different marine scientists discussing various topics of marine science.	Biweekly on Fridays
BIMS Bites Kids	The second in our BIMS TV series, these are 3-minute episodes with Andrew Walker discussing various topics of marine science.	Monthly on Saturdays
BIMS Dives	The third in our BIMS TV series, these are 60-minute Q&As with prominent marine scientists discussing various topics of marine science with different BIMS members.	Last Friday of the month
BIMS Hall	The fourth in our BIMS TV series, these are 60-minute Q&As with the CEO of BIMs.	Third Tuesday of the month
BIMS Reads	The fifth in our BIMS TV series, these are short book readings of various authors who write about the ocean.	Quarterly
BIP Week	Week of events to introduce minority undergrads to marine science, includes SCUBA certification, marine lab tours, and hands-on field experience.	Annually
BIMS Week	Week of events to amplify the voices of black marine scientists, includes workshops, panels, and keynotes by BIMS members across the globe.	Annually
Paid Internships	BIMS has partnered with the NOAA to provide an immersive science communication experience and The Nature Conservancy & Doris Duke to provide a field research experience.	Annually
Stem Hands Camp	A marine science camp for deaf minority youth.	Annually
BlueGAP	A collaborative research project aimed to educate frontline communities on nutrient pollution while amplifying environmental champions voices through a documentary and online platform.	Ongoing

With the growth of BIMS TV, our social media pages and press coverage, we developed BIMS memberships to create a database of black marine scientists around the globe and develop a community that had been missing from the field. We believe in providing paid internships and speaking engagements to our members while also protecting them against predatory recruitment strategies. We ask for transparency and honesty from everyone who interacts with BIMS members or else they get that privilege revoked; we are protecting this space at all costs. Because of that, we have launched our ally membership and organizational partnerships, which allow allies and organizations to financially support BIMS memberships and provide access to our ally safe space chat and posting on our job board. We need people to put their money where their mouth is and becoming a BIMS ally member shows action. Allies need to be willing to speak up against injustices but also hold their colleagues accountable for creating safe workspaces. With the BIMS ally member network, we are working together to share successes and creating our own best practices to build a blueprint for cultural change.

We currently have over 300 members in 27 different countries and are expanding more every day. Outside of BIMS Week and BIMS TV, we have launched several programs (Table 1) like our Black in Marine Science Immersion Program (BIP Week), which provides undergraduates from minority communities with a SCUBA certification and immerses them in various types of hands-on marine science training (e.g., coral restoration, shark tagging, science communication). I have had the opportunity to travel the world and share the mission of BIMS in places like Egypt and Portugal for the UN Decade of Ocean Science, while also creating safe spaces for BIMS members to network at conferences like Capitol Hill Ocean Week and The American Fisheries Society. We have also been doing outreach in the community—black communities most impacted by climate change such as sea level rise and nutrient pollution—educating them and also getting more youth excited about becoming marine scientists. Our BIMS Bites Kids series is dedicated to showing black youth that they can be what they can see and preparing all kids to expand their idea of what a scientist looks like from a young age. Lastly, we have made our content and outreach accessible to our deaf colleagues by partnering with Vidman Barber to provide American Sign Language for all our BIMS TV content. When I say diversity in marine science, I mean everybody.

My dream for BIMS and diversifying the field is to build The BIMS Institute. It will be the largest black-led marine science research institution in a community where black people actually live, dedicated to innovative research and community engagement. When I think of US marine science institutions, not only are the majority of the employees white, so are the communities in which they are based, causing isolation and potential racism at every turn. I was a full-time scientist living in Seattle, but it did not stop my neighbors from slamming the door to our building in my face, assuming I did not live there. These experiences inside and outside of the lab are enough to make people want to leave the field completely, so it is time for us to create the space we need.

I have noticed that in many diversity programs and initiatives, there is a lack of understanding of the true needs of black people. Yes, I want to be invited to present my research at a conference, but if you are going to ask us to travel to remote and potentially hostile locations, with no safety plan in place or even thought about, then leave us out of it. What nay-sayers need to understand is that we are not asking for any favors. We just want the people who claim they want us in these spaces to be held accountable for making these rooms impossible to thrive in. Conferences, departments, and any workspaces that go out of their way to invite black people in need to prepare the environment. We want to believe that everyone is onboard with every diversity initiative, but the truth is they are not. To combat this, we must include departmental trainings and visits in our funding requests to ensure we are providing the best conditions for all our future scientists, because ultimately we need more scientists to solve the current conditions harming our ocean. We should not have to worry about racism and toxic environments when the ocean has literally been on fire.

At the end of the day, we are marine scientists. We want our voices to be heard [5], we want our work to be published and cited [6], we want to be invited to give scientific talks, elected to leadership boards and to have our research funded at the highest levels, and we will. We have had varied levels of support since founding BIMS. Some allies have been sincere while others have simply jumped on the bandwagon. To the performers, I need you to do better. To my real allies, thank you from the bottom of my heart, we honestly could not do this work without you. To my BIMS community, thank you for the opportunity to serve. I am sorry we have had to endure so much pain and trauma, but rest assured, a new wave is here!

## References

[1] Langin K. ‘A time of reckoning.’ How scientists confronted anti-Black racism and built community in 2020. Science. 2020 [cited 2022 Sep 13] Available from: https://www.science.org/content/article/time-reckoning-how-scientists-confronted-anti-black-racism-and-built-community-2020.

[2] Bernard RE, Cooperdock EHG. No progress on diversity in 40 years. Nat Geosci. 2018;11:292–295. doi: 10.1038/s41561-018-0116-6

[3] Moore T. The only Black person in the room. Limnol Oceanogr Bull. 2018;27:114–115. doi: 10.1002/lob.10269

[4] Black In Marine Science. [Internet] Available from: https://www.youtube.com/blackinmarinescience.

[5] Gaynus C. A statement from the Black woman in the room. Limnol Oceanogr Bull. 2020;29:75–75. doi: 10.1002/lob.10384

[6] El-Sabaawi R, Kantar MB, Moore T, Pantel JH, Tseng M, Ware J. The EEB POC project. Limnol Oceanogr Bull. 2020;29:97–99. doi: 10.1002/lob.10390

